# Contextual and structural representation learning for fine-grained barefoot footprint region recognition

**DOI:** 10.3389/fbioe.2026.1912110

**Published:** 2026-10-14

**Authors:** Yujie Shen, Wenxin Xie, Yabin Zhao, Xuemei Jiang, Jianrong Wang

**Affiliations:** 1 College of Investigation, People’s Public Security University of China, Beijing, China; 2 Trace Examination Technology Department, Institute of Forensic Science of China, Beijing, China; 3 School of Automation and Software Engineering, Shanxi University, Taiyuan, China

**Keywords:** barefoot footprint recognition, context awareness, multi-scale fusion, spatial feature reconstruction, YOLOv10

## Abstract

Barefoot footprints provide valuable biometric evidence for forensic investigation and suspect identification. However, the rich structural details and diverse scale distributions of footprint images pose significant challenges for accurate fine-grained region recognition. Existing methods often struggle to effectively model contextual information and structural characteristics, limiting their ability to characterize subtle morphological variations. To address these issues, this paper proposes a robust framework for contextual–structural representation learning in barefoot footprint region recognition. A context-guided spatial feature reconstruction pyramid network (CGSFRPN) is introduced to enhance semantic consistency and feature interactions across different scales. To improve directional perception and edge detail modeling, a Direction-Aware Pinwheel Convolution (DAPConv) is incorporated to capture structural patterns with diverse orientations. Furthermore, a ConvFormer module based on the MetaFormer architecture is employed to facilitate efficient semantic representation and cross-region information integration. By jointly exploiting contextual information and structural characteristics, the proposed framework achieves robust recognition of fine-grained footprint regions, including toes, arches, and heels. Experiments conducted on a proprietary barefoot footprint dataset demonstrate that the proposed method achieves a mean Average Precision (mAP) of 83.2%, outperforming several mainstream object detection approaches and exhibiting superior visualization performance. These results highlight the effectiveness and robustness of the proposed framework for fine-grained barefoot footprint analysis and its potential applications in intelligent forensic identification.

## 1 Introduction

As a unique and highly recognizable biological feature, barefoot footprints have long played an important role in crime scene investigation and suspect identification (Wang K. et al., 2024). Barefoot footprints can not only reflect the physiological characteristics of an individual, but also reveal their walking style, body habits and even movement trajectory, providing richer behavioral clues. Therefore, accurate detection and identification of barefoot footprints are of irreplaceable significance for restoring the crime scene and narrowing the scope of investigation (Nagwanshi, 2022). Traditional footprint recognition methods rely on manually designed feature extraction technology, which is difficult to fully capture the fine-grained information with discriminative value in footprints, which leads to low recognition accuracy (Yang Y. et al., 2025).

In recent years, the rapid development of deep learning technology has provided new solutions for footprint recognition. Image recognition models based on convolutional neural networks (CNNs) have significantly improved the accuracy and robustness of recognition (Li et al., 2020). In particular, single-stage target detection frameworks, such as the YOLO (You Only Look Once) series. This type of method has high inference speed and good detection performance, and has been widely used in fields such as autonomous driving and industrial defect detection. YOLOv10 (Wang A. et al., 2024) is a highly efficient version of this series. It optimizes the decoding structure and multi-scale feature fusion mechanism, effectively improving the target detection effect and capability while maintaining a low computational overhead. However, due to the special structure of footprint images and their complex spatial distribution characteristics, existing target detection methods often find it difficult to fully capture the axial semantic information and multi-scale features of footprints, which leads to low recognition accuracy (Nagwanshi and Dubey, 2018). To address the above issues, this paper proposes a model for footprint key region detection based on the YOLOv10 architecture—CDCNet. The model integrates the CGSFRPN, DAPConv and ConvFormer modules to effectively enhance the structural expression and key region modeling capabilities. Specifically, CGSFRPN combines pyramid context extraction (PCE) with rectangular self-calibration module (RCM) to improve structural modeling and axial expression capabilities. We designed a multi-branch feature fusion module (FFM) and a dynamic interpolation fusion (DIF) module to enhance semantic alignment and scale adaptability. The DAPConv we constructed captures footprint edge and directional features through multi-directional asymmetric convolutions, and ConvFormer achieves efficient cross-region semantic modeling and multi-scale fusion.

The main contributions of this paper are as follows.

1. To address the problem of complex structural details and difficulty in accurately modeling key regions in footprint images, we propose a structure-aware footprint key region detection network CDCNet. It can integrate context modeling, structure reconstruction and scale enhancement mechanisms to effectively improve the detection performance of footprint key regions.
2. To solve the problems of insufficient structural expression and difficulty in multi-scale alignment, we designed a context-guided spatial feature reconstruction module CGSFRPN. It integrates PCE, RCM, FFM and DIF to achieve structural continuity modeling and scale adaptive fusion.
3. To address the problems of insufficient directional feature extraction and weak semantic modeling capabilities, we constructed a DAPConv and ConvFormer module. It can improve directional perception and cross-region semantic consistency respectively, and strengthen the feature expression effect of key structures.

This paper is organized as follows: Section 2 describes the related research work on footprint recognition. Section 3 introduces the CDCNet model proposed in this paper in detail. Section 4 conducts experimental analysis and discussion. Section 5 summarizes the paper and looks forward to future research directions.

## 2 Related work

Existing footprint recognition methods are divided into two categories: traditional footprint recognition methods and footprint recognition methods based on deep learning.

### 2.1 Traditional footprint recognition methods

Traditional footprint recognition methods achieve matching and discrimination between individuals by modeling information such as footprint contour, regional shape, and geometric proportion. For example, Liu et al. (2023) proposed a barefoot footprint similarity measurement method based on image binarization and edge extraction. Gao et al. (2020) constructed a footprint model based on three-dimensional reconstruction technology, which can extract the regional relationship features and shape length features of the footprint, and combine it with principal component analysis to achieve identity discrimination. Khokher and singh (2016) analyzed the role of foot length and width features in identity recognition and verified the effect of geometric features on person discrimination. Nakajima et al. (2000) combined the regional information in the footprint image with the posture variable to achieve person recognition under multi-source features.

Although traditional methods have achieved footprint recognition to a certain extent, they rely on the design and extraction of artificial features and have the following two problems: First, they rely on manual feature design rules, and they are difficult to extract feature information such as the shape and scale of different footprints. Second, they focus on local geometric properties and cannot explore the structural associations and semantic relationships between key regions such as the big toe, arch and heel.

### 2.2 Deep learning footprint recognition method

Since convolutional neural networks have the ability to automatically extract features, deep learning-based methods for identifying key regions of footprints have developed rapidly. For example, Wang et al. (2019) combined the deep features extracted by CNN with geometric information and realized footprint recognition through support vector machines. Bao et al. (2020) constructed a spatially aggregated weighted convolutional neural network (SAW-CNN), which effectively improved footprint recognition under occlusion conditions. Zhu et al. (2020) proposed a multi-scale self-attention mechanism that can extract structural details of footprints. Zhu et al. (2023) constructed a deep metric learning network based on triplet loss, which effectively enhanced the ability to extract discriminative and clustering information. Jin et al. (2022) proposed a multi-scale feature fusion network to improve the model’s sensitivity to structures such as toes and arches, thereby improving the recognition accuracy of footprints. Bao et al. (2022) constructed a non-local attention dual-branch network to achieve cross-modal retrieval of optical and pressure footprints. Wu et al. (2024) proposed a cross-modal footprint image retrieval algorithm that integrates shape and multi-level information to address the modal difference between grayscale footprint images and dust footprint images. Park et al. (2022) estimated the foot position information of footprint shadow images based on an improved Fast R-CNN network. Chen et al. (2021) proposed an integrated deep neural network model based on transfer learning to improve the retrieval efficiency of footprint images. Zhang et al. (2025) proposed a footprint image segmentation method based on multi-granular features and regional relationships, and strengthened global feature modeling through a spatial relationship matrix.

Existing footprint recognition methods have made some progress in feature extraction and cross-modal feature fusion, but there are still challenges in the representation and semantic fusion of key information such as toes, arches and heels. To this end, we propose a CDCNet model that integrates contextual guidance with pyramid feature perception. It improves recognition performance for key regions through structure-guided encoding and multi-scale feature fusion. This model takes into account the requirements of efficiency and accuracy, and provides technical support for the application of footprint recognition in actual scenarios.

## 3 Proposed model

### 3.1 Overall framework

We propose a CDCNet for footprint key region detection, whose overall architecture is shown in Figure 1. CDCNet maintains the original YOLOv10m detection framework while introducing targeted modifications to enhance directional feature perception, contextual semantic modeling, and multi-scale feature representation. Specifically, DAPConv is incorporated into the backbone by replacing the corresponding standard convolutional layers, enabling the network to extract orientation-sensitive local features during hierarchical feature extraction. ConvFormer is further employed to replace the corresponding C2f and C2fCIB blocks in the backbone and detection path, thereby enhancing local feature extraction and channel-wise semantic modeling. During backbone feature extraction, multi-scale feature maps P3, P4, and P5 are generated at different resolutions. These feature maps are subsequently processed by CGSFRPN, which reconstructs the original multi-scale feature fusion pathway through PCE, RCM, FFM, and DIF. PCE first aggregates contextual information from P3, P4, and P5 to construct a unified multi-scale contextual representation, while RCM performs spatial self-calibration and contextual reconstruction. FFM and DIF then progressively fuse and align features across different spatial resolutions before forwarding the reconstructed representations to the subsequent detection path. The resulting multi-scale features are further processed by the feature extraction blocks containing ConvFormer and are finally transmitted to the detection head for classification and localization of the eight footprint region categories.

**FIGURE 1:**
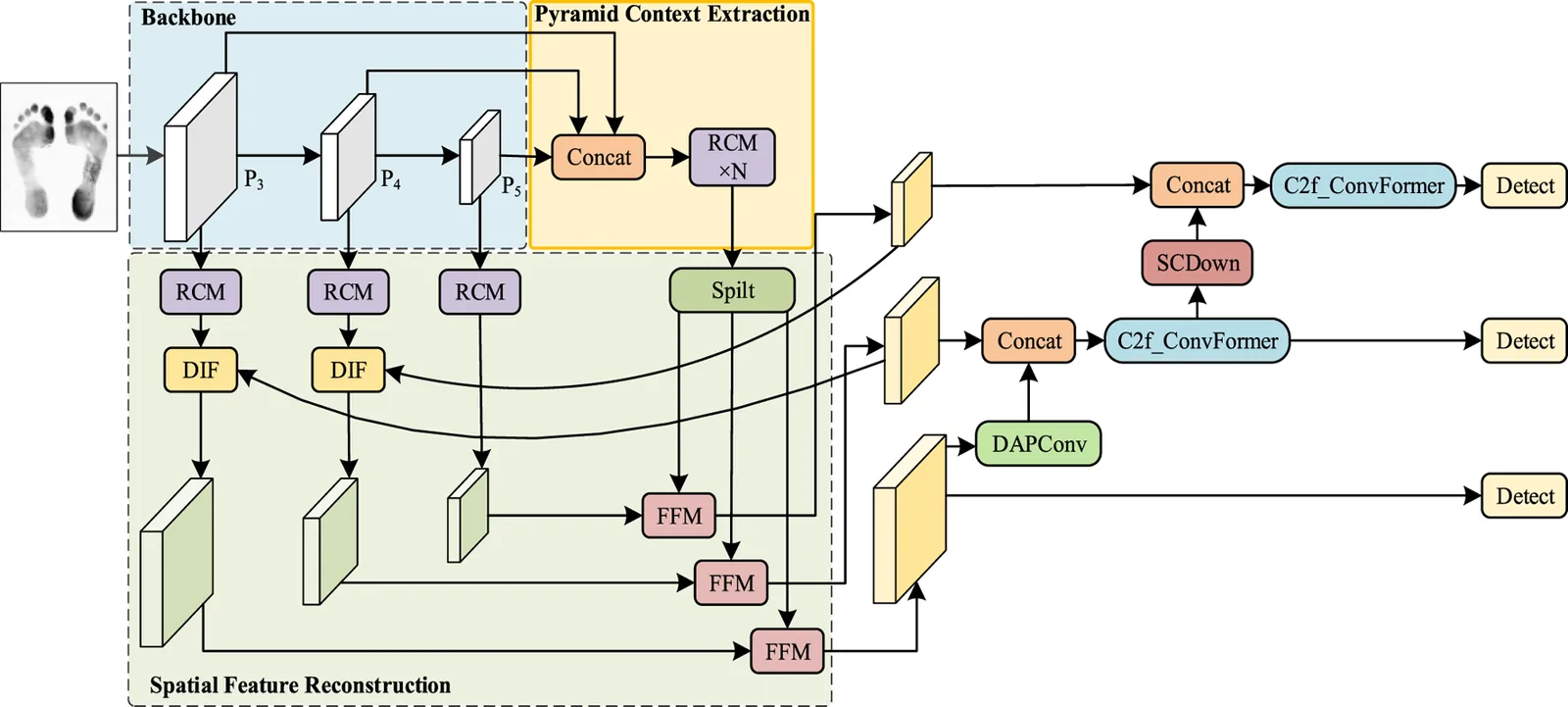
Overall structure of CDCNet.

To further clarify the structural relationship between CDCNet and the YOLOv10m baseline, the proposed modules are integrated through targeted replacement and reconstruction of the corresponding baseline components rather than by simply adding independent processing branches. DAPConv replaces the corresponding standard convolutional layers in the backbone, while ConvFormer replaces the designated C2f and C2fCIB feature extraction blocks in both the backbone and detection path. CGSFRPN reconstructs the original multi-scale feature fusion pathway by integrating PCE, RCM, FFM, and DIF. Through this organization, the backbone first extracts hierarchical directional and semantic representations, from which P3, P4, and P5 are obtained and subsequently reconstructed and fused by CGSFRPN. The resulting multi-scale representations are then propagated to the detection path, where ConvFormer-based feature extraction further enhances the semantic representation before the final detection head performs classification and localization. Therefore, CDCNet forms a coherent feature-processing pipeline in which DAPConv, ConvFormer, and CGSFRPN perform complementary functions in directional feature extraction, semantic representation learning, and contextual multi-scale feature reconstruction, respectively.

### 3.2 Context-guided spatial feature reconstruction pyramid network

To improve the model’s ability to understand key regions in footprint images, we propose a CGSFRPN. This module’s spatial feature reconstruction mechanism models the feature map’s spatial dimensions (Ni et al., 2024) and integrates multi-scale contextual information to reconstruct features with both directional and regional awareness. Specifically, we introduce the RCM and the PCE module. RCM and PCE perform axial modeling of the feature map horizontally and vertically and also fuse multi-layer semantic information contextually. We also design a multi-branch FFM and a DIF to achieve spatial alignment and cross-scale information reconstruction, effectively improving perception of key regions such as toe length, arch shape, and heel structure. Thus, CGSFRPN not only maintains detailed local modeling but also enhances contextual semantic integration, providing more discriminative features for footprint recognition.

#### 3.2.1 Rectangular self-calibration module

The RCM captures global contextual information in horizontal and vertical directions via axial feature modeling and enhances the representation of rectangular structures. This module improves spatial representation of key footprint regions, strengthens the model’s perception of footprint orientation and layout, and mitigates the lack of local feature detail. The structure of RCM is shown in Figure 2 and includes Rectangular Self-Calibration Attention (RCA), Batch Normalization (BN), and a Multilayer Perceptron (MLP). Let the input feature of RCM be $x\in R^{H\times W\times C}$ , where H , *W*, and *C* denote the height, width, and number of channels of the input feature map, respectively.

**FIGURE 2:**
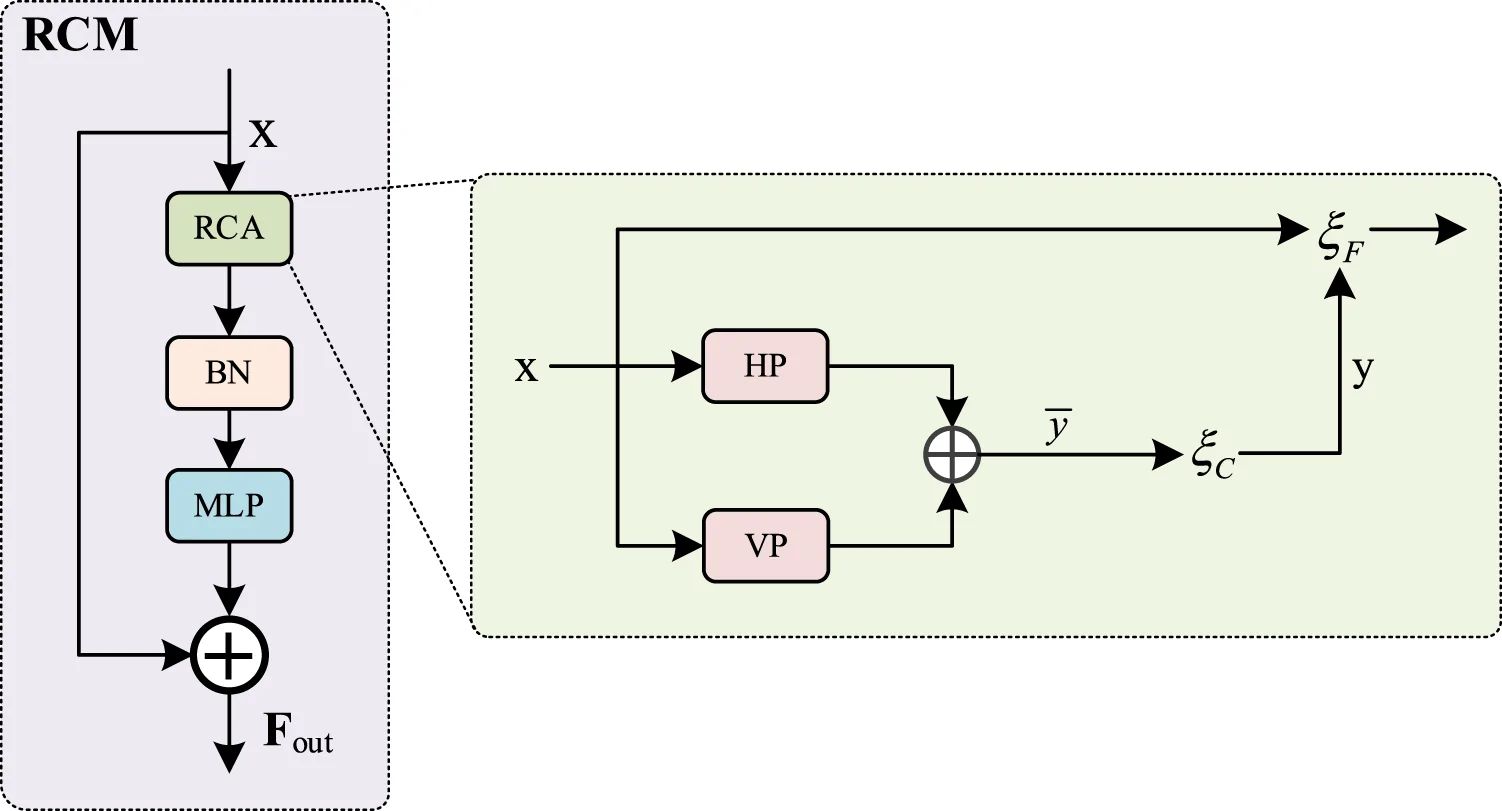
RCM module structure diagram.

First, RCA captures the global context information of the image in the horizontal and vertical directions through horizontal pooling (HP) and vertical pooling (VP), respectively, and generates two axial feature vectors. Then, these two vectors are broadcasted and added to form a rectangular region of interest, denoted as $\bar{y}$ . The resulting feature $\bar{y}$ has the same spatial and channel dimensions as the input feature. In order to make the generated rectangular attention region closer to the actual shape of the target, RCA introduces a shape self-calibration function. This function calibrates the attention region in the horizontal and vertical directions through two large-kernel strip convolutions (Yuan et al., 2026). Among them, the horizontal strip convolution adjusts each row element to make the horizontal shape closer to the foreground target. The vertical strip convolution adjusts each column element to make the vertical shape closer to the foreground target. The shape self-calibration function calculation formula is:

$$\xi_{C}\left(\bar{y}\right)=\delta \left(\psi_{k\times 1}\left(\phi \left(\psi_{1\times k}\left(\bar{y}\right)\right)\right)\right)$$ (1)

where *ψ* represents large kernel strip convolution. *k* is the kernel size of strip convolution. *ϕ* represents the combined operation of batch normalization and ReLU activation function. δ is the Sigmoid activation function, which generally takes a value between 0 and 1. The first 1×k strip convolution performs horizontal feature calibration, while the subsequent k×1 strip convolution performs vertical feature calibration. The output $\xi_{C}\left(\bar{y}\right)$ has the same dimension as $\bar{y}$ , i.e., $\xi_{C}\left(\bar{y}\right)\in R^{H\times W\times C}$ .

In order to fuse the calibrated attention features with the original input features, RCA designed a feature fusion function. It can further extract the local details of the input features through 3 × 3 deep convolution, and then use the Hadamard product to weight the calibrated attention features to the input features. The calculation formula of the feature fusion function is:

$$\xi_{F}\left(x,y\right)=\psi_{3\times 3}\left(x\right)⊙y$$ (2)

where $\psi_{3\times 3}$ represents a 3 × 3 depthwise convolution. *y* is the attention feature after being processed by the shape self-calibration function. ⊙ represents the Hadamard product, namely, element-wise multiplication. Since the two feature maps have the same dimensions, the output $\xi_{F}\left(x,y\right)$ also has the dimension $R^{H\times W\times C}$ .

Then, the output of RCM is transformed nonlinearly through BN and MLP layers, and the calculation formula is:

$$F_{\text{out}}=\rho \left(\xi_{F}\left(x,\xi_{C}\left(\text{HP}\left(x\right)⊕\text{VP}\left(x\right)\right)\right)\right)+x$$ (3)

where ⊕ represents broadcast addition. HP and VP represent horizontal pooling and vertical pooling, respectively. ρ represents batch normalization (BN) and multi-layer perceptron (MLP). Specifically, HP and VP first aggregate the input feature *x* along the horizontal and vertical directions, respectively. The resulting features are then combined by broadcast addition and processed by the shape self-calibration function $\xi_{C}\left(\cdot \right)$ . The calibrated feature is subsequently fused with the input feature through $\xi_{F}\left(\cdot \right)$ , followed by the nonlinear transformation ρ . Finally, a residual connection is used to add the original input *x*, producing the output $F_{\text{out}}\in R^{H\times W\times C}$ . Through these operations, RCM can effectively capture global context information and enhance the model’s ability to perceive the key regional features of the footprint.

Through the above design, RCM not only improves the model’s modeling ability for target regions with rectangular structures and enhances its perception of directionality and spatial relationships, but also enables CGSFRPN to achieve a better balance between structural modeling and contextual information extraction.

#### 3.2.2 Pyramid context extraction module

PCE constructs a cross-level feature pyramid to integrate contextual information from different feature scales. This design is particularly relevant to footprint region detection because different anatomical regions exhibit substantial variations in spatial extent and morphological detail. For example, toe-related structures are relatively small and detail-sensitive, whereas arch and heel regions generally occupy larger areas and contain broader structural patterns. By aggregating features from P3, P4, and P5 into a unified contextual representation, PCE facilitates the interaction between fine-grained local details and high-level structural semantics.

The PCE module structure is shown in the yellow box in Figure 1. Specifically, the PCE model is embedded in the YOLOv10 backbone network, which can perform context reconstruction and fusion of multi-scale feature maps *P*_3_, *P*_4_, and *P*_5_. These three feature maps correspond to 1/8, 1/16, and 1/32 downsampling of the input image, respectively. Assuming that the input image size is *H* × *W*, the spatial dimensions of *P*_3_, *P*_4_, and *P*_5_ are *H*/8×*W*/8, *H*/16×*W*/16, and *H*/32×*W*/32, respectively. PCE downsamples the feature maps of these three scales to a uniform size (i.e., 1/64 scale of the original image) through average pooling. They are spliced together to generate the pyramid feature P6, and the calculation formula is as follows:

$$P_{6}=\text{Concat}\left(\text{AP}\left(P_{3},8\right),\text{AP}\left(P_{4},4\right),\text{AP}\left(P_{5},2\right)\right)$$ (4)

where AP(*P*,x) denotes the average pooling operation that reduces the spatial resolution of feature map *P* by a factor of *x*. Specifically, AP(*P*_3_,8), AP(*P*_4_,4), and AP(*P*_5_,2) transform the three feature maps into the same spatial resolution of *H*/64×*W*/64. Concat (⋅) represents channel-wise concatenation. This step realizes the effective fusion of features of different scales and forms a pyramid feature representation of multi-scale information.

Then, the PCE module interactively fuses the pyramid feature *P*_6_ through three stacked RCM. The output of each RCM block is sequentially fed into the next RCM block to progressively enhance contextual representation. The calculation formula is as follows:

$$P_{\text{out}}=\text{RCM}\left(P_{6}\right)$$ (5)

where *P*_6_ denotes the input pyramid feature generated by multi-scale feature aggregation, and *P*_out_ represents the output feature after contextual reconstruction by stacked RCM blocks.

Through the combination of cross-level aggregation and progressive contextual reconstruction, PCE enables the model to jointly exploit fine-grained and high-level structural information. The RCM blocks further provide horizontal and vertical structural responses, helping the model preserve geometric relationships within footprint regions and improving the representation of morphological characteristics across different spatial scales.

#### 3.2.3 Multi-scale feature fusion mechanism

The multi-scale feature fusion mechanism in CGSFRPN consists of two complementary components, namely, the multi-branch FFM and the DIF. They are organized as an alternating fusion and alignment pathway to connect the spatially calibrated features from RCM with the contextual features generated by PCE. FFM performs semantic-aware feature fusion, while DIF dynamically aligns the fused features across spatial resolutions. Specifically, FFM fuses feature maps of different resolutions through convolution operations and element-wise multiplication. Its structure is shown in Figure 3. The input of FFM includes: feature maps processed by the RCM module (denoted as *x*_1_), and semantic features of the same scale output by the PCE module (denoted as *x*_2_). In the deepest level (such as *P*_5_), *x*_1_ comes from the backbone network. In the shallow layer (such as *P*_4_, *P*_3_), *x*_1_ comes from the output of the previous level (such as *P*_5_, *P*_4_) after FFM and DIF fusion. *x*_1_ and *x*_2_ pass through the convolution layer to extract local features respectively, and the convolution output of *x*_2_ generates a channel attention map through H-sigmoid activation, which can achieve semantic enhancement. Then, *x*_2_ is interpolated to the same size as *x*_1_, and the channel is weighted through the attention map. Finally, the weighted feature is element-wise multiplied with the convolution result of *x*_1_ to generate a fused feature map. This process realizes context-aware feature reconstruction and effectively improves the consistency and discriminability of structural expression.

**FIGURE 3:**
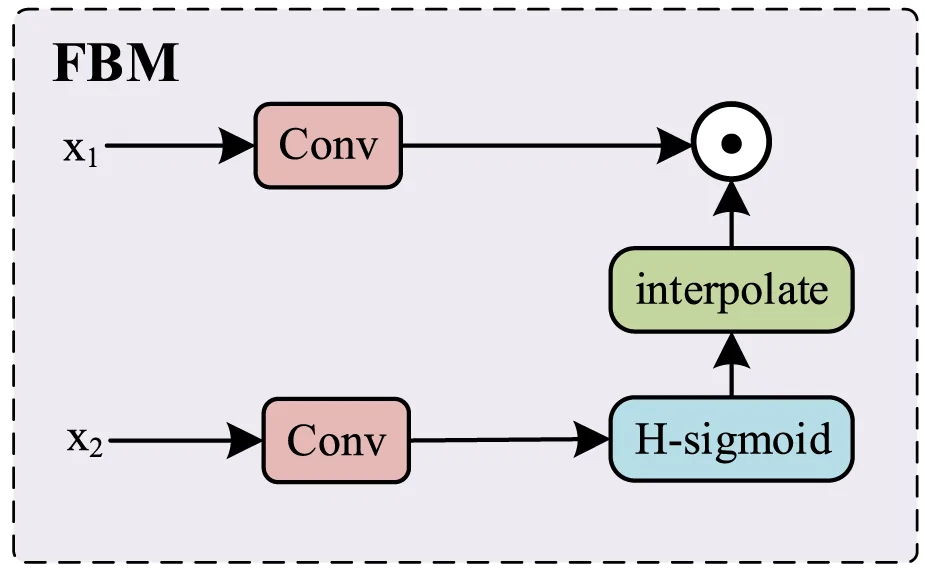
FFM structure diagram.

The DIF structure is shown in Figure 4. DIF receives the feature *x*_1_ processed by RCM and the fused feature *x*_2_ from the output of the lower-layer FFM. For the smaller feature map *x*_2_, it is first interpolated to have the same resolution as *x*_1_, then features are extracted by convolution, and finally added to *x*_1_ to obtain the fused result after dynamic alignment.

**FIGURE 4:**
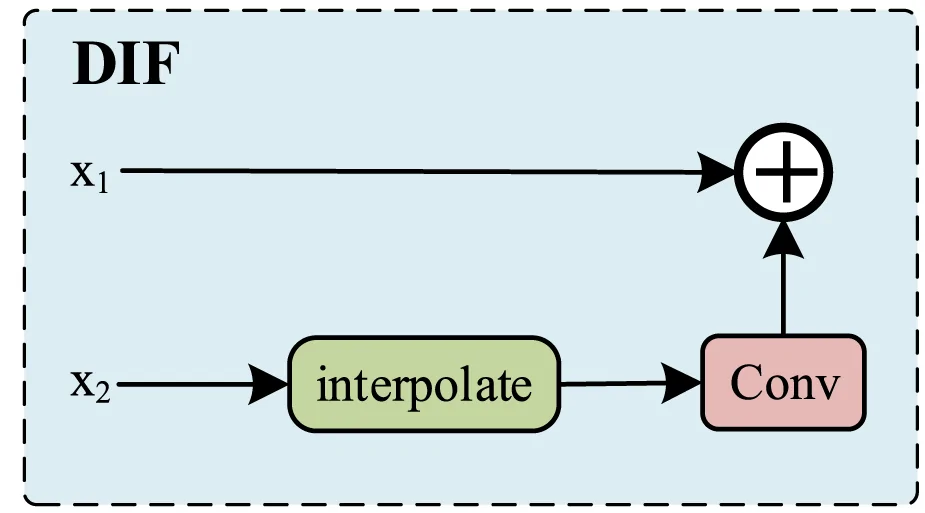
DIF structure diagram.

The layer-by-layer alternation of FFM and DIF enables the model to progressively integrate backbone features with contextual semantic information while maintaining cross-scale feature consistency. This design is particularly suitable for footprint region detection because the target regions exhibit substantial differences in spatial scale and morphological appearance. For example, toe-related regions contain relatively fine local structures, whereas arch and heel regions occupy broader spatial areas. Direct fusion of features from different levels may weaken fine structural details or introduce spatial misalignment during cross-scale propagation. FFM therefore performs semantic-aware fusion between spatial and contextual features, while DIF further aligns features with different spatial resolutions before their propagation to subsequent levels. Through this alternating fusion and alignment process, CGSFRPN can preserve fine boundary and contour information while incorporating broader contextual cues, which is beneficial for distinguishing subtle morphological differences among toe, arch, and heel regions.

### 3.3 Direction-aware pinwheel convolution module

Barefoot footprint regions contain abundant directional structural information. In particular, toe boundaries, arch contours, and heel outlines exhibit different orientations, local curvatures, and elongated geometric patterns (Li et al., 2024). These directional characteristics are important for distinguishing fine-grained morphological categories, such as different toe-length patterns and arch or heel shapes. However, conventional convolution mainly aggregates local information within a regular receptive field and may not sufficiently emphasize orientation-specific boundary responses. To address this issue, we incorporate the DAPConv into the feature extraction stages of CDCNet to strengthen directional structural representation. Rather than simply adding an independent feature-processing branch, DAPConv is used to replace the corresponding standard convolutional layers in the YOLOv10m backbone, allowing directional feature extraction to be integrated directly into the hierarchical representation learning process.

DAPConv introduces convolution branches with directional selectivity. By using asymmetric padding and multi-directional sampling, it expands the receptive field and guides each convolution to focus on structural boundaries (Yang J. et al., 2025). Specifically, assuming that the input tensor is *X*∈*R* ^*H*×*W*×*C*^, where *H*, *W*, and *C* denote the height, width, and channel number of the input feature map, respectively, DAPConv designs four groups of directional convolution branches. Moreover, it uses asymmetric padding and 1 × 3 or 3 × 1 convolution kernels to extract features in different spatial directions. Four directional branches are constructed to model horizontal and vertical structural information. Each branch adopts different padding strategies to enhance the perception of directional boundaries. The specific calculation formula is as follows:

$$\begin{array}{c} X_{1}\left(h^{\prime },w^{\prime },c^{\prime }\right)=SiLU\left(BN\left(X_{P\left(1,0,0,3\right)}⊗W_{1}^{\left(1,3,c^{\prime }\right)}\right)\right)\\ X_{2}\left(h^{\prime },w^{\prime },c^{\prime }\right)=SiLU\left(BN\left(X_{P\left(1,0,0,3\right)}⊗W_{1}^{\left(3,1,c^{\prime }\right)}\right)\right)\\ \begin{array}{c} X_{3}\left(h^{\prime },w^{\prime },c^{\prime }\right)=SiLU\left(BN\left(X_{P\left(0,1,3,0\right)}⊗W_{3}^{\left(1,3,c^{\prime }\right)}\right)\right)\\ X_{4}\left(h^{\prime },w^{\prime },c^{\prime }\right)=SiLU\left(BN\left(X_{P\left(3,0,1,0\right)}⊗W_{4}^{\left(3,1,c^{\prime }\right)}\right)\right) \end{array} \end{array}$$ (6)

where *P* (*a*,*b*,*c*,*d*) represents the number of padded pixels in the up, down, left, and right directions. ⊗ represents the convolution operation. *W*_*i*_ is the directional convolution kernel. Specifically, $W_{1}^{\left(1,3,c^{\prime }\right)}$ and $W_{3}^{\left(1,3,c^{\prime }\right)}$ are 1 × 3 convolution kernels with c′ output channels, while $W_{2}^{\left(3,1,c^{\prime }\right)}$ and $W_{4}^{\left(3,1,c^{\prime }\right)}$ are 3 × 1 convolution kernels with *c*′ output channels. *h*′, *w*′, and *c*′ denote the spatial positions and channel index of the output feature maps. BN is batch normalization. SiLU is the activation function.

After directional features are extracted, the output feature maps of the four branches are concatenated in the channel dimension to form a tensor. The specific calculation formula is:

$$X^{\prime }\left(h^{\prime },w^{\prime },4c^{\prime }\right)=Cconcat\left(X_{1},X_{2},X_{3},X_{4}\right)$$ (7)

where, *X*′ denotes the fused directional feature representation, and the channel dimension is increased to 4*c*′ because four directional features are concatenated. Then, a 2 × 2 convolution is used for dimensionality reduction and channel fusion, and the calculation formula is:

$$Y\left(h_{2},w_{2},c_{2}\right)=SiLU\left(BN\left(X^{\prime }⊗W^{\left(2,2,c_{2}\right)}\right)\right)$$ (8)

where *W* ^(2, 2, c2)^ represents the 2 × 2 convolution kernel used for channel reduction and feature fusion. *h*_2_, *w*_2_, *c*_2_ denote the spatial positions and channel index of the final output feature map *Y*.

For footprint region detection, the directional representation provided by DAPConv is particularly useful for preserving boundary and contour information. The boundaries of toe-related regions are often elongated along specific directions, while arch and heel regions contain curved or approximately linear contour segments with varying orientations. Therefore, the directional branches can provide complementary responses to these local geometric patterns, helping the network distinguish regions with similar overall appearance but different boundary shapes or orientations. This is especially relevant to fine-grained footprint recognition, where category differences may depend on subtle changes in toe length, arch curvature, or heel contour rather than on large differences in global appearance.

Through this directional feature extraction process, DAPConv provides orientation-sensitive local representations while maintaining compatibility with the hierarchical convolutional structure of YOLOv10m. Its integration into the corresponding feature extraction stages enables CDCNet to complement the contextual and multi-scale representations provided by CGSFRPN, forming a more task-oriented representation of footprint regions.

### 3.4 ConvFormer module

Based on the MetaFormer (Yu et al., 2024) architecture, we incorporate a ConvFormer module into CDCNet, as shown in Figure 5. ConvFormer combines depthwise separable convolution (Chollet, 2017) with a channel MLP. In CDCNet, the ConvFormer module replaces the corresponding C2f and C2fCIB feature extraction blocks in the YOLOv10m backbone and detection path, providing convolution-based token mixing and channel-wise semantic modeling for subsequent feature reconstruction and fusion.

**FIGURE 5:**
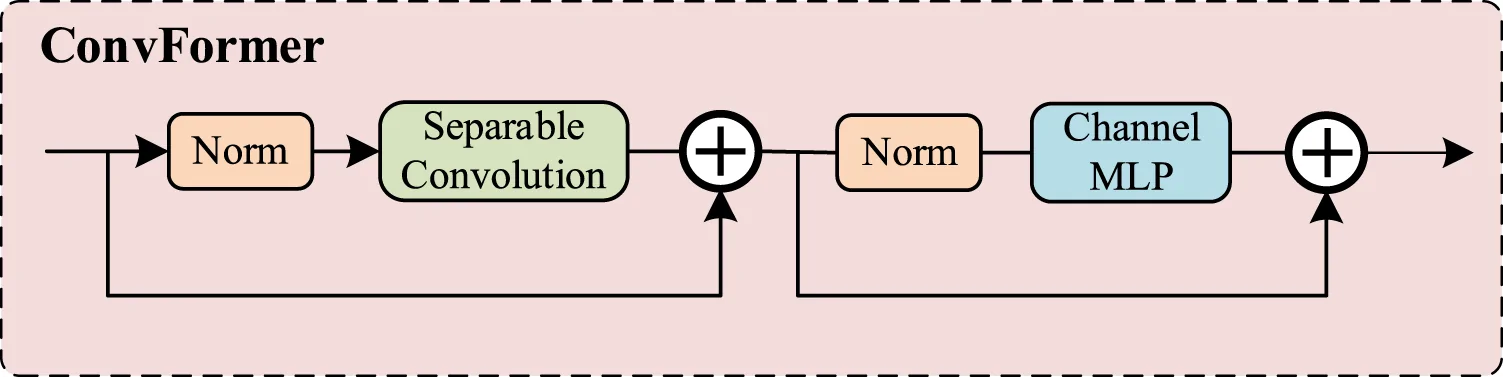
ConvFormer module structure diagram.

Given input feature *X∈ R* ^*H×W×C*^, where *H*, *W*, and *C* denote the height, width, and number of channels of the feature map, respectively, it is first normalized to obtain the normalized feature *X*_norm1_. Then, the normalized feature is input into the separable convolution module. This module adopts the structure of “point-by-point convolution-depth-by-depth convolution-point-by-point convolution”, which can efficiently separate spatial and channel information. The specific calculation formula is as follows:

$$Y=\text{Convpw}2\left(\text{Convdw}\left(\sigma \left(\text{Convpw}1\left(X_{norm1}\right)\right)\right)\right)$$ (9)

Here, Convpw1 (·) represents the first point-wise convolution with a kernel size of 1 × 1, which expands the channel dimension of the input feature from *C* to an intermediate dimension *C*′. Convdw (·) represents the depth-wise convolution, where each channel is convolved independently with a spatial kernel to extract local spatial information while keeping the channel dimension unchanged. Convpw2 (·) denotes the second point-wise convolution, which projects the feature dimension from *C*′ back to the original channel dimension *C*. $\sigma \left(.\right)$ represents the nonlinear activation function.

Then, the output *Y* of the separable convolution is connected with the input feature *X* through residual connection to obtain feature *Z*. This method can effectively alleviate the gradient vanishing and promote information flow. *Z* is normalized and input to the multi-layer perceptron module. This module models the relationship between channels through nonlinear mapping and enhances the global dependency expression ability of features. Finally, the output of the multi-layer perceptron module is fused with the input *Z* through residual connection to obtain a stronger feature expression. This combination is beneficial for footprint region recognition because it preserves fine local structures while strengthening semantic relationships among feature channels, which is important for distinguishing footprint regions with similar appearances but different morphological characteristics. ConvFormer uses the StarReLU activation function, and the calculation formula is as follows:

$$\text{StarReLU}\left(x\right)=s\cdot {\left(\text{ReLU}\left(x\right)\right)}^{2}+b$$ (10)

where *s* is a learnable scaling factor. *b* is a learnable bias term. StarReLU introduces square terms and adjustable parameters, which not only improves the nonlinear representation ability, but also enhances the model’s ability to respond to local and global features.

ConvFormer maintains the computational efficiency of the YOLOv10m framework while enhancing local-to-semantic feature representation. This property is particularly beneficial for footprint region recognition, where different regions exhibit distinct spatial extents and morphological characteristics. Fine structures in toe-related regions require detailed local feature extraction, whereas arch and heel regions rely more on contextual semantic information. By combining convolution-based local feature extraction with channel-wise semantic modeling, ConvFormer can capture both fine-grained structural details and higher-level semantic information, thereby improving the representation of footprint regions with different morphological characteristics.

## 4 Experiments and analysis

### 4.1 Footprint dataset

The proposed CDCNet is evaluated on a self-built barefoot footprint dataset containing 2,587 high-resolution ink-print footprint images collected from 573 different individuals. Unlike conventional footprint recognition tasks that generally perform global classification on complete footprint images, this study focuses on the detection and analysis of fine-grained morphological regions within barefoot footprints. Therefore, the task is formulated as a fine-grained footprint region detection problem based on an object detection framework. By predicting the bounding boxes and corresponding category labels of specific regions, the proposed method is capable of automatically locating and identifying key anatomical regions and their morphological characteristics in complete footprint images.

In this study, the footprint images were collected using the ink-print acquisition method. Ink-printing is a traditional and widely used footprint acquisition technique, in which ink is evenly applied to the plantar surface and the participant leaves a footprint impression on recording paper. The acquisition procedure was conducted on a hard and flat indoor surface. High-quality Xuan paper with good ink absorption and mechanical strength was used as the recording medium to preserve clear plantar structures. Commercial carbon-based ink was appropriately diluted and evenly distributed over the plantar surface using a sponge applicator. Before acquisition, each participant cleaned and dried their feet to ensure uniform ink adhesion. Participants then sequentially performed footprint collection under the guidance of experimental personnel. After each acquisition, the footprint impressions were inspected for completeness and clarity. Samples with blurred impressions, incomplete plantar structures, or severe overlap were excluded or recollected. Finally, each valid footprint sample was indexed and recorded, and the paper-based impressions were digitized using a high-resolution scanner for subsequent annotation and analysis.

All footprint images were manually annotated by experienced annotators, and all annotations were subsequently reviewed by another researcher to ensure labeling consistency. Unlike conventional object detection tasks in which the targets are independent physical objects, the detection targets in this study are fine-grained anatomical regions and morphological attributes within the same complete barefoot footprint. Therefore, bounding-box annotations were used to locate the corresponding anatomical regions, and each annotated region was assigned one category label according to its morphological characteristic. As illustrated in Figure 6, each complete barefoot footprint is anatomically divided into three major regions: the forefoot, midfoot, and rearfoot. The eight detection categories are defined based on the morphological characteristics of these three regions. Specifically, the forefoot contains two toe-related categories, namely, thumb-toe length and second-toe length, representing the morphological characteristics of the first and second toes, respectively. The midfoot contains three arch-type categories, including high arch, narrow arch, and flat arch, which are distinguished according to the contact area and contour characteristics of the arch region. The rearfoot contains three heel-shape categories, namely, round heel, oval heel, and rectangular heel, which are defined according to the contour shape of the heel region. Therefore, the eight categories correspond to morphological attributes of the three anatomical regions rather than eight independent objects distributed across the footprint. The correspondence between the anatomical regions and the eight fine-grained categories is summarized as follows: the forefoot includes thumb-toe length and second-toe length; the midfoot includes high arch, narrow arch, and flat arch; and the rearfoot includes round heel, oval heel, and rectangular heel. Each bounding box is annotated within its corresponding anatomical region, and the detailed examples of the eight categories are presented in Figure 7.

**FIGURE 6:**
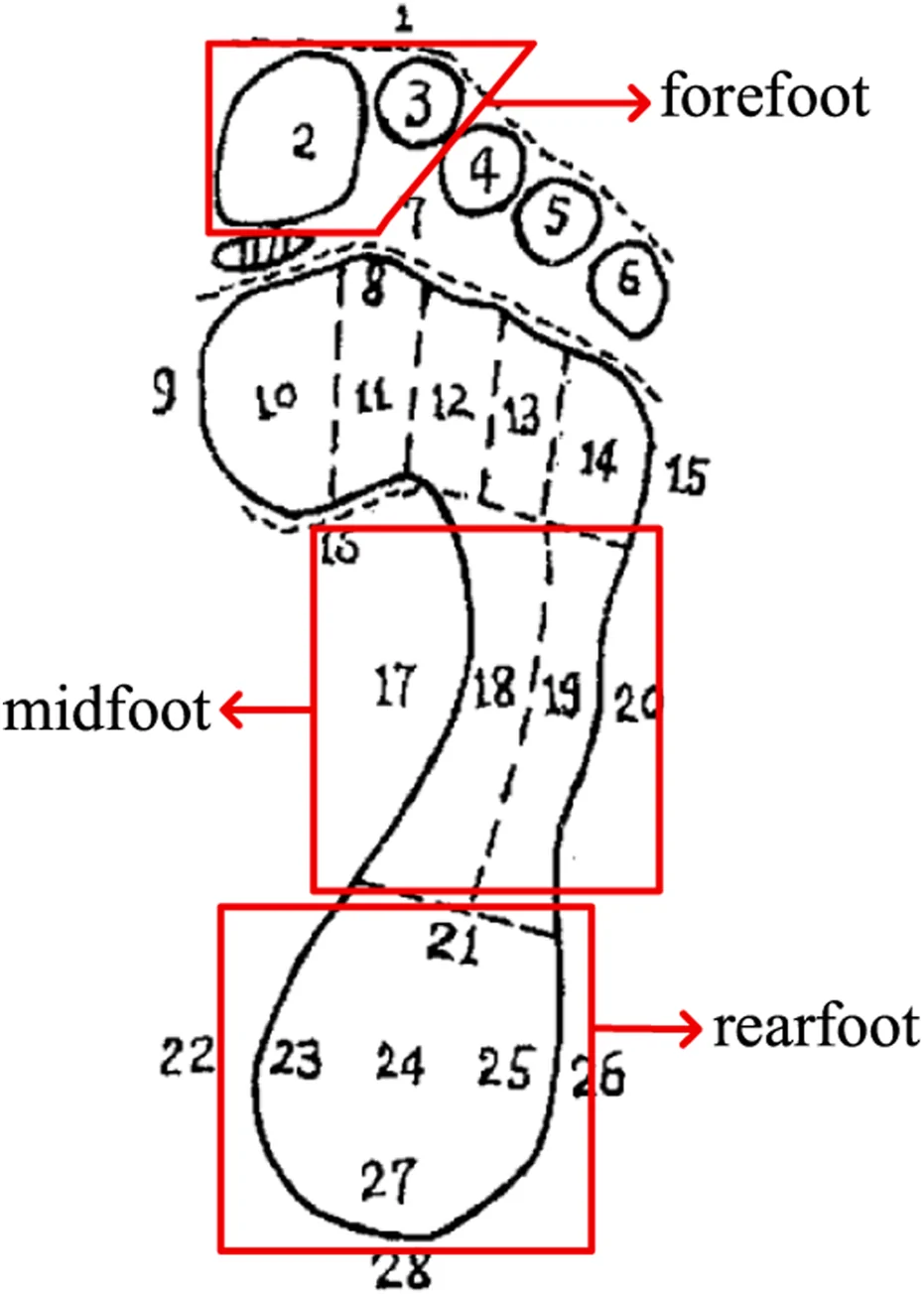
Three anatomical regions of a barefoot footprint.

**FIGURE 7:**
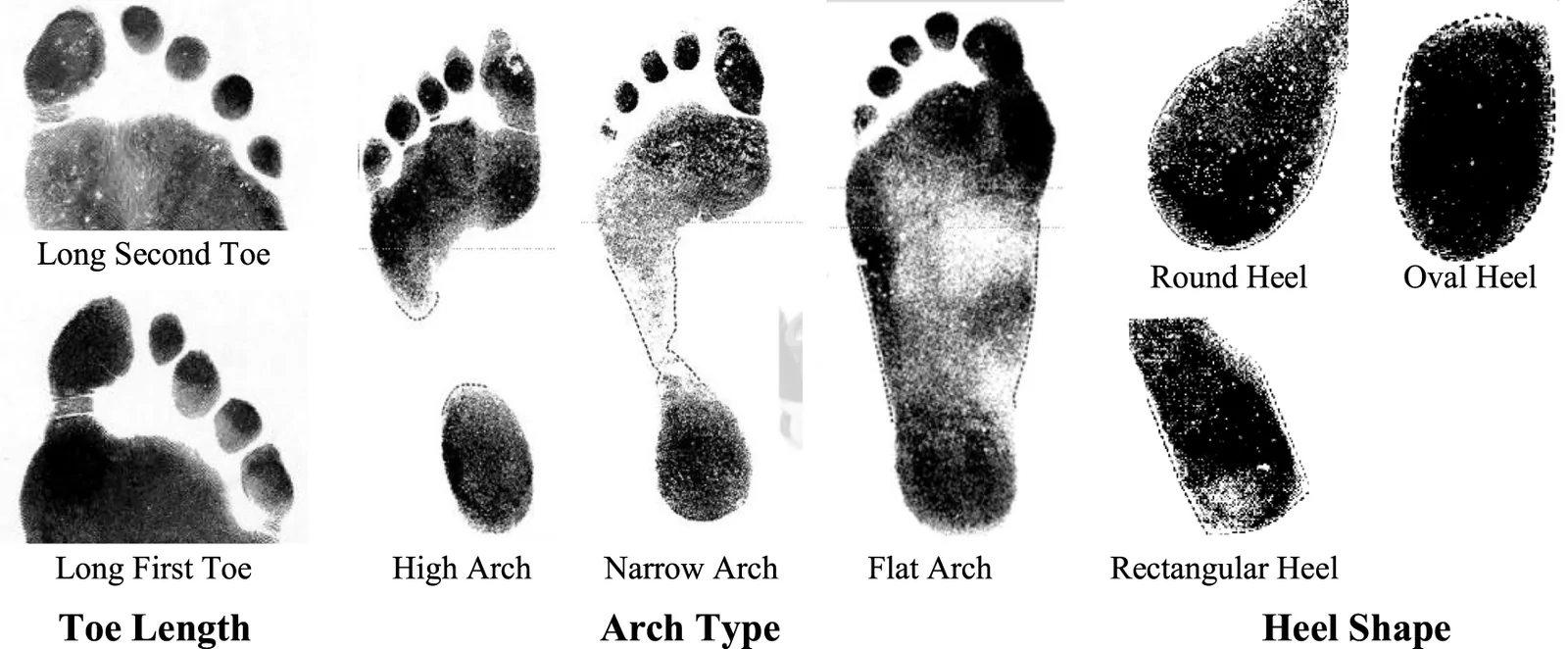
Eight footprint categories.

For model training and performance evaluation, the 2,587 footprint images were divided into training, validation, and testing subsets at a ratio of 7:2:1, resulting in 1,810, 518, and 259 images, respectively. Representative examples of the collected barefoot footprint images are shown in Figure 8. Importantly, the dataset partition was performed at the subject level rather than at the image level. Specifically, all footprint images collected from the same individual were assigned exclusively to one subset, and no individual contributed samples to more than one of the training, validation, or testing subsets. This subject-disjoint partition strategy prevents identity leakage between different subsets and reduces the possibility that the model benefits from subject-specific characteristics during evaluation. Consequently, the testing set contains only footprints from individuals who are not present in the training or validation sets, providing a more reliable assessment of the model’s generalization capability. The resulting dataset covers diverse plantar morphological variations across individuals and provides reliable annotations for fine-grained footprint region detection.

**FIGURE 8:**
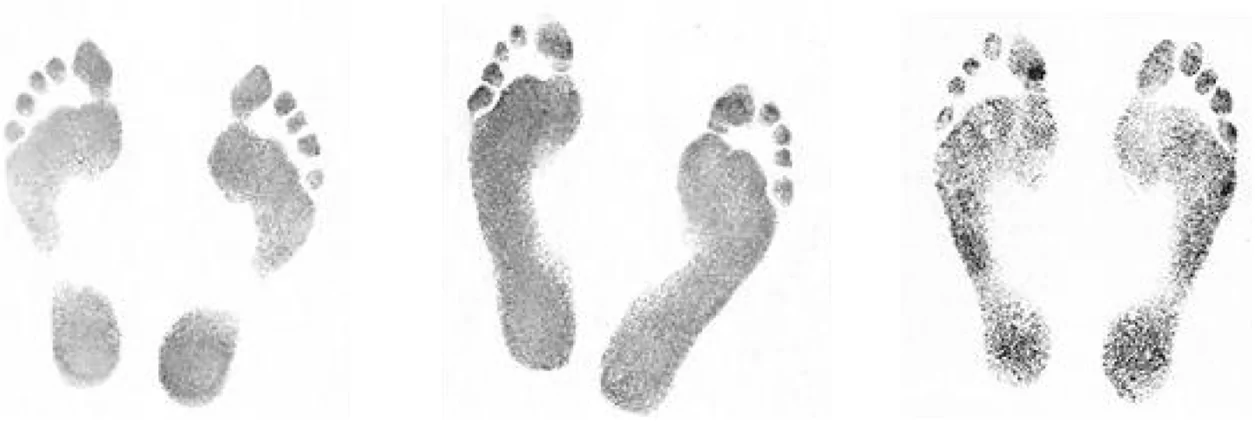
Barefoot footprint sample.

### 4.2 Experimental environment setting and evaluation indicators

All experiments were conducted on Ubuntu 20.04 with an NVIDIA L40 GPU and an Intel® Xeon® Platinum 6330 CPU. To ensure a fair comparison and reproducibility, all detection models were re-trained on the same footprint dataset under identical experimental settings with a fixed random seed. Specifically, all models used the same dataset partition, input resolution, training epochs, and optimization configuration.

The dataset was divided into training, validation, and testing subsets with a ratio of 7:2:1, and all compared methods were trained and evaluated using the same data samples. During training, all input images were resized to 640 × 640 pixels, and the same data augmentation strategy was applied to all models. Since all compared models were trained from scratch without using any external pre-trained weights, all methods were optimized directly on the footprint dataset. All models were trained for 200 epochs using the SGD optimizer with the same batch size and optimization strategy. No additional hyperparameter optimization was performed for individual models, ensuring that the performance differences mainly reflect the feature representation capability of each method. Training parameters are listed in Table 1.

**TABLE 1:** Training parameter settings.

Name	Value
Image size	640
Batch size	16
Epochs	200
Optimizer	SGD

We use Precision (P), Recall (R), mAP@0.5, mAP@0.5:0.95, and GFLOPs to evaluate performance. P measures the proportion of predicted positives that are correct, R measures the proportion of actual positives detected, mAP@0.5 is the average precision at IoU = 0.5, mAP@0.5:0.95 is average over IoU 0.5–0.95, and GFLOPs indicates inference complexity.

### 4.3 Ablation experiment

To comprehensively evaluate the contribution of each proposed component, we conduct ablation experiments based on the YOLOv10m baseline. The progressive construction of CDCNet follows the functional hierarchy of the three modules. CGSFRPN is introduced first because it reconstructs and integrates multi-scale contextual features, providing a stronger feature representation for subsequent processing. DAPConv is then incorporated to enhance the directional perception of local boundaries and contour structures on the basis of the reconstructed features. Finally, ConvFormer is introduced to strengthen local-to-global semantic modeling and channel-wise feature interactions, thereby further improving the representation of fine-grained footprint regions. This progressive order reflects the complementary roles of contextual reconstruction, directional feature extraction, and semantic modeling, and allows the contribution of each component and their combined effects to be evaluated progressively. In addition to the progressive combinations, each module is also evaluated independently to analyze its individual contribution. All experiments use the same dataset and experimental environment. The results are presented in Table 2. (“✓” indicates inclusion of a module, and “-” indicates absence).

**TABLE 2:** Ablation experiment results.

Sequence	CGSFRPN	DAPConv	ConvFormer	P (%)	R (%)	mAP@0.5(%)	mAP@0.5:0.95(%)	Gflops
1	-	-	-	69.3	78.7	78.0	54.6	58.9
2	✓	-	-	70.7	77.0	79.2	55.7	59.0
3	-	✓	-	72.2	75.9	78.4	55.4	61.3
4	-	-	✓	72.0	79.9	81.8	58.7	54.7
5	✓	✓	-	73.7	79.1	81.1	58.3	61.5
6	✓	-	✓	73.8	80.8	82.2	58.3	52.1
7	-	✓	✓	69.8	82.2	80.9	57.1	57.1
8	✓	✓	✓	77.8	78.1	83.2	59.6	54.6

The YOLOv10m baseline achieves an mAP@0.5 of 78.0% and an mAP@0.5:0.95 of 54.6%. After introducing CGSFRPN alone, the mAP@0.5 increases to 79.2%, demonstrating that the contextual feature reconstruction mechanism effectively improves the perception of structural regions such as toe and arch areas. This indicates that multi-scale contextual aggregation helps the model capture discriminative footprint characteristics.

When DAPConv is independently introduced, the model achieves an mAP@0.5 of 78.4% and an mAP@0.5:0.95 of 55.4%. Although the improvement in mAP@0.5 is limited, the increase in mAP@0.5:0.95 indicates that DAPConv enhances the localization capability of fine boundary structures by improving directional feature perception.

The independent use of ConvFormer improves mAP@0.5 from 78.0% to 81.8%, confirming that global semantic modeling is beneficial for distinguishing footprint regions with similar appearances. Furthermore, combining two modules consistently improves detection performance. For example, the combination of CGSFRPN and ConvFormer achieves 82.2% mAP@0.5, while the combination of CGSFRPN and DAPConv obtains 81.1% mAP@0.5.

Finally, the complete CDCNet integrating all three modules achieves the best performance, with mAP@0.5 of 83.2% and mAP@0.5:0.95 of 59.6%. Compared with the YOLOv10m baseline, CDCNet improves mAP@0.5 and mAP@0.5:0.95 by 5.2% and 5.0%, respectively. Notably, the complete CDCNet achieves a lower computational cost of 54.6 GFLOPs than the YOLOv10m baseline (58.9 GFLOPs), demonstrating that the proposed modules collectively improve detection accuracy while maintaining computational efficiency.

In summary, each module contributes: CGSFRPN deepens feature perception and semantic aggregation; DAPConv enhances boundary recognition; and ConvFormer uses global modeling and attention for better semantics. Together, they improve accuracy on complex footprints while keeping resource use low.

### 4.4 Comparative experiment

We compare CDCNet with representative detectors, including Faster R-CNN (Ren et al., 2017), YOLOv8 (Yaseen, 2024), YOLOv10 (Wang A. et al., 2024), YOLOv12 (Tian et al., 2025), RT-DETR (Zhao et al., 2023), and the lightweight Mamba-YOLO (Wang Z. et al., 2024). To ensure a fair comparison, all methods are trained and evaluated using the same experimental protocol described in Section 3.2. Specifically, all models use the same dataset partition, input resolution, data augmentation strategy, training epochs, batch size, optimizer, and optimization settings. All models are trained from scratch without using external pre-trained weights, and no model-specific hyperparameter optimization is performed. The comparative results are presented in Table 3.

**TABLE 3:** Comparative experimental results.

Types	Faster R-CNN	YOLOv8m	YOLOv10m	RTDETR	YOLOv12m	Mamba-YOLO	Ours
mAP@0.5	48.13	77.8	78.0	69.6	80.4	81.2	**83.2**
mAP@0.5:0.95	34.87	54.7	54.6	49.2	57.7	57.3	**59.6**

The bold values represent the values with the highest accuracy.

Faster R-CNN is a classic two-stage detector, and its mAP@0.5 and mAP@0.5:0.95 are 48.13% and 34.87% respectively. The recognition accuracy of this method is low, because the Faster R-CNN method does not adequately model the fine-grained features of the footprint image.

YOLOv8 is a typical method in the YOLO series, and its mAP@0.5 is 77.8% and mAP@0.5:0.95 is 54.7%. This method has been significantly improved over Faster R-CNN. This is because it can extract small difference regions of the footprint image (such as toe length and arch edge), thereby achieving better recognition accuracy.

YOLOv10 is lightweight and structurally optimized based on YOLOv8. It obtains a mAP@0.5 of 78.0% and a mAP@0.5:0.95 of 54.6%. The recognition accuracy of this method is better than that of the YOLOv8 method. This is because YOLOv10 has designed more efficient feature extraction and fusion modules.

YOLOv12 introduces a context-guided mechanism based on the previous version, further improving the model’s recognition ability. It achieves an mAP@0.5 of 80.4% and an mAP@0.5:0.95 of 57.7%. This is because it can locate complex structures and fuzzy boundary regions such as arches and heels, and can effectively distinguish footprint features with high structural similarity.

RT-DETR belongs to the lightweight model of the DETR series. The mAP@0.5 and mAP@0.5:0.95 obtained by this method are 69.6% and 49.2% respectively. Although the recognition accuracy it obtains is better than Faster R-CNN, it still has a certain gap with the YOLO series algorithms. This is because RT-DETR is more sensitive to the size of training samples, and the global modeling has a low dependence on local features, making it difficult to effectively identify subtle feature regions in footprint images.

Mamba-YOLO also achieved high recognition accuracy, with an mAP@0.5 of 81.2% and an mAP@0.5:0.95 of 57.3%.

Our method achieves the highest accuracy: 83.2%/59.6%. Compared to the best existing methods (Mamba-YOLO and YOLOv12), our mAP@0.5:0.95 is about 2.3% and 1.9% higher, respectively. This is because CGSFRPN enhances contextual modeling, DAPConv improves directional feature representation, and ConvFormer provides stronger global perception. Together, they make the model highly robust to complex footprint regions (e.g., toe gaps, arch boundaries, heel contours).

To further illustrate performance, Figure 9 visualizes detection results from RT-DETR, YOLOv12, Mamba-YOLO, and CDCNet at confidence thresholds 0.5 and 0.3. At 0.5 confidence, RT-DETR misses parts like the “long thumb” and “oval” heel of the right foot, and its boxes poorly match contours. YOLOv12 misses the right toe. Mamba-YOLO misses the “long thumb” and “oval” left heel, and mislabels the right foot’s “narrow arch” as “flat arch,” showing it struggles with subtle morphology. These methods suffer from background interference in complex regions, causing false or missed detections. Lowering confidence to 0.3 causes YOLOv12 and Mamba-YOLO to produce overlapping boxes with blurred boundaries, indicating unstable filtering. RT-DETR, YOLOv12, and Mamba-YOLO all exhibit false detections at 0.3.

**FIGURE 9:**
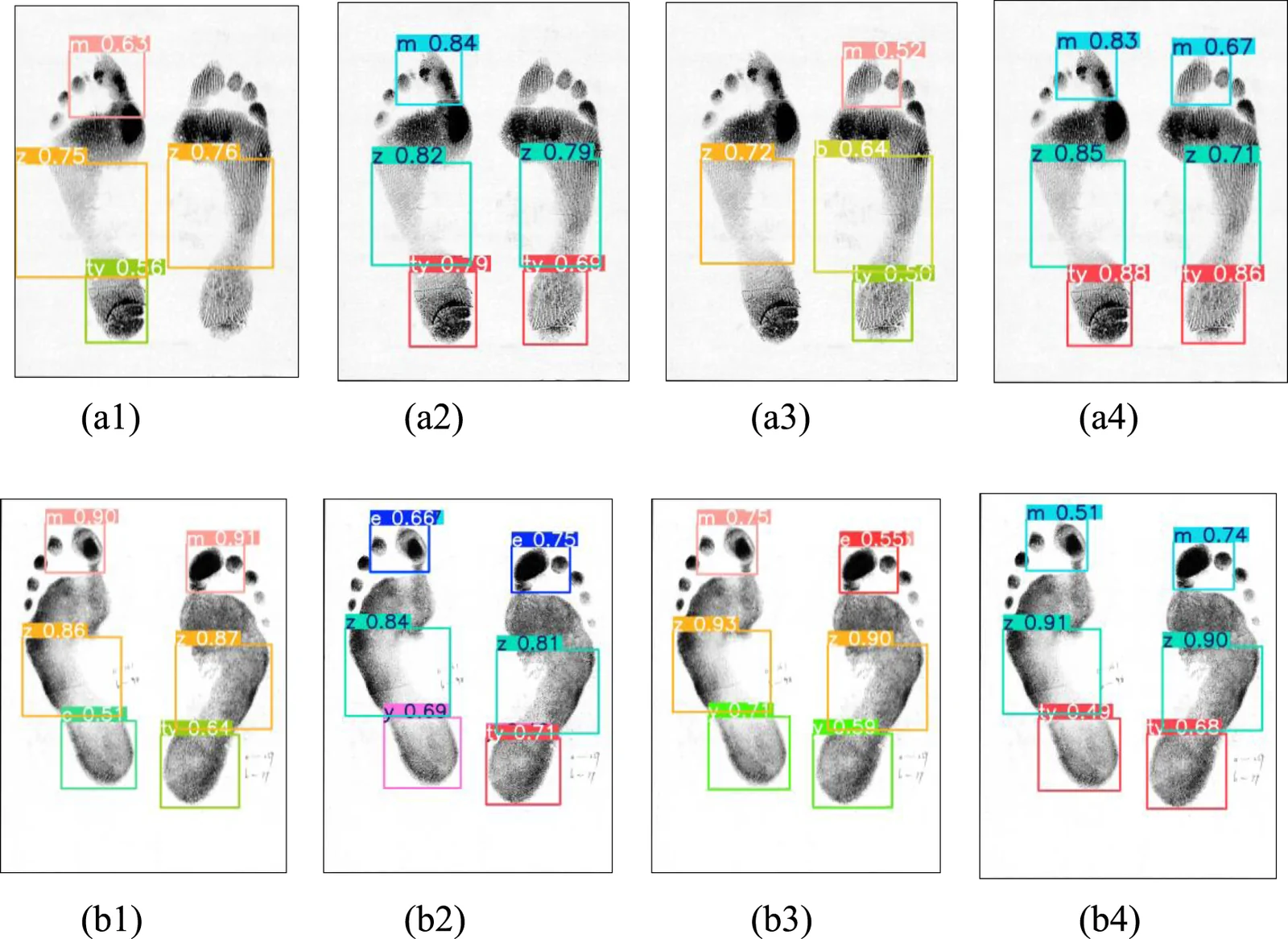
Visualization results of different models **(a1–a4)** show detection results of RT-DETR, YOLOv12, Mamba-YOLO, and CDCNet at confidence 0.5. **(b1–b4)** Show the same at confidence 0.3. Letters indicate key region categories: e (two-finger length), m (thumb length), g (high arch), z (narrow arch), b (flat arch), y (round heel), ty (elliptical heel), c (rectangular heel).

In contrast, CDCNet excels at both confidence levels. At 0.5, CDCNet accurately identifies key regions (toe length, arch type, heel shape); boxes are precisely placed with clear outlines and no missed detections or category confusion. At 0.3, CDCNet has fewer misses, and results remain stable without false detections or offsets.

## 5 Conclusion

We propose an improved detection model CDCNet based on the YOLOv10 architecture, to address the challenge of identifying key regions in barefoot footprint images. We design a CGSFRPN that integrates spatial reconstruction with pyramid context fusion, significantly improving structural modeling of key regions such as toe length, arch, and heel. We also introduce DAPConv, which enhances directional perception and improves footprint feature extraction. Our ConvFormer module enhances the combination of global semantic modeling and local feature extraction. Ablation and comparative experiments on our footprint dataset show that CDCNet outperforms mainstream methods in metrics like mAP@0.5 and mAP@0.5:0.95, achieving a good balance between accuracy and efficiency. Visualization results further demonstrate the model’s ability to capture complex footprint morphology: CDCNet accurately identifies key regions like toe length, arch type, and heel shape with no obvious missed detections or confusion.

Future work will focus on improving the model’s generalization across scenarios, enabling deployment in complex environments and edge devices, and broadening its applications in related fields.

## Data Availability

The original contributions presented in the study are included in the article/supplementary material, further inquiries can be directed to the corresponding authors.
